# The Senile Cardio-Vascular System

**Published:** 1907-11-16

**Authors:** T. Clifford Allbutt

**Affiliations:** K.C.B.


### Hospital Clinics.

### THE SENILE CARDIO-VASCULAR SYSTEM.

By PROFESSOR T. CLIFFORD ALLBUTT, K.C.B., M.D., F.R.S.

The subject of my lecture is that of senile cardio-arterial disease, and it is one about which there is so much to be said that my notes are for selection rather than comprehension. First let us consider the adjective "senile." The last time I was lecturing at Cambridge I was discussing the case of a man aged forty-one, and I had reason to suspect some difference between my audience and myself regarding his age. My class looked upon the man as already old; I, on the other hand, regarded him as still youthful.

Now, for our purposes of the moment, we may roughly divide the ages of man into three. We may speak of the period from zero to thirty as the age of acute infections; that from thirty to sixty we may take as the period of chronic infections, and maladies such as gout, cancer, diabetes, etc.; from sixty or sixty-five to ninety we are comparatively free from chronic infections (except, as we are assured, those arising from our own colons) and enter an age of slowly advancing senility, or preparation for death. In respect of arterial disease, I have of late used the word "decrescence" for this period, and the corresponding adjective "decrescent." I used to call it the "involutionary" period, but this word was criticised, and perhaps justly, on the ground that in old age not every man becomes atheromatous. The corresponding word *decrementum* is used in respect of senile processes by Haller. To-night we are to consider the first two age periods only in so far as they sow the seeds of chronic maladies in the third.

It is with some surprise that I find arterio-sclerosis in use as a chapter heading in recent medical works. It is still called *a* disease, and to-day I have been reading a paper by an able author on the "treatment of arterio-sclerosis." Now arterio-sclerosis is a pathological result; not a disease but a result of many diseases, any of which may, singly or together, be the cause of it. Furthermore, as I said at Toronto, even from a strictly pathological point of view it is not a single condition; it is certainly dual, probably multiform. It is true that under the microscope, in terminal stages, it is difficult to draw distinctions between the different forms; the artery is now in ruin. It is then very difficult to say exactly along which path the artery had come to grief. But I think the pathologists will admit that in earlier stages we may distinguish at least two processes, probably more. As a clinical term the word has no business to be used at all; its application should be purely pathological. Therefore, while to consider arterio-sclerosis as a uniform pathological process may lead us into some confusion, if we speak of it clinically as a disease we shall get into still worse confusion.

Disease of the arteries has, of course, been known for a very long time. I cannot now enter upon the history of the matter, but I will refer you to the pages of Haller, who described arterio-sclerosis as well as anyone could do who was not provided with modern instruments of research. Coming to more recent times, I think the debt we owe to von Basch, of Vienna, whose decease, a short time ago, we had to deplore, is very imperfectly recognised. His work has been served up over and over again by his successors, sometimes with, but often without, acknowledgment. Too often I find von Basch's teaching copied verbally out of his works without any indication of its source. Now, von Basch took the view that arterio-sclerosis, arising from whatever cause, produces directly a gradual increase of blood-pressure, and is the chief cause of this increase; this he tried to explain on physical grounds. Far back in the eighties some of us had pointed out that blood-pressures do not always rise in arterio-sclerosis; and that pressures frequently do rise in cases apparently free from this change. Von Basch replied that in these cases the arterio-sclerosis might exist in a latent form—that is, confined to the mesenteric arteries, or to some other region where we have no means of revealing it—and this notion is not incredible.

Meanwhile Burdon Sanderson, Mahomed, Huchard and myself were working at blood-pressures with imperfect instruments, but I was not aware of this part of Huchard's work until after I read my paper to the Hunterian Society in 1894.* Independently Huchard and I then parted from von Basch so far as to regard the arterio-sclerosis as rather a consequence than a cause of enhanced blood-pressures. Huchard, however, did not carry his mechanical principles into the explanation of the cardiac failure. Instead of regarding the heart as failing after a struggle against increasing work, he spoke of cardio-sclerosis as a co-ordinate condition. In Huchard's school the matter stood thus—that arterio-sclerosis is a mechanical condition arising from excessive blood-pressure, the degeneration of the arteries being due to overstretching.

But in 1894 I pointed out again that this is not absolutely the case; for in many persons there is arterio-sclerosis without undue blood-pressure, or only with that increase of blood-pressure which is normal or usual in many people over fifty. For some years I was alone in this opinion, and I think Groedel, of Nauheim, was the first to corroborate it; Hasenfeld, Strassburger, and others have verified it since. Groedel demonstrated that in 25 per cent. of the cases of arterio-sclerosis the blood-pressure is not raised above this quasi-normal standard of the elderly. Arterio-sclerosis of itself does not raise the blood-pressure considerably; it is something else that causes this rise, and in 1894 I classified patients with arterio-sclerosis in three kinds:—

First, those with pre-eminently high pressure;

* I have quoted this paper as 1894, the year in which it was written; but I see it was not read to the Society till February 1895.

I have called this Hyperpiesis; the increase is here permanent and morbid, and not "senile" in nature.

Second, the toxic cases, in which there is usually no increase of pressure, not infrequently a decrease. Thus it is common enough, say in cases of diabetes in girls of twelve or fourteen, to find arteries as thick as those of a man of sixty, but yet under very moderate blood-pressures. I am often surprised that these facts have attracted so little attention. The increased thickness may in these patients be merely a muscular increase, as it may be in young people after the infectious diseases. Thayer has described it as a sequel of typhoid fever, when it is not uncommon and may be transitory. So also syphilis and other conditions may cause it in such patients; yet the blood-pressure is very often low. There are exceptions to this rule about blood-pressure in toxic states, as in the case of lead poisoning, when the pressure is high; here, however, the condition of the kidney may be the disturbing element.

The decrescent or senile arterio-sclerotic cases belong to my third class. At this point it is convenient to say a word or two about the kidneys. Granular kidney, as I am always urging upon my pupils, is an improper word to use as a description of a pathological naked-eye appearance. In this sense every organ in an old man is more or less "granular." If in such a case there are granular casts in the urine you are justified in supposing the kidneys to be "granular," even without seeing them; but that is a different story. The name "granular kidney" is accepted as a clinical name, as the name of a certain disease; it must not be applied merely to the appearance of a kidney. The essential point is, What is the state of its secretory tubules? In the arterio-sclerotic kidney of old people there are large islands of normal tubules in the kidney, interspersed among areas of obliterated tubules. This was well illustrated in a microphotograph of a so-called granular kidney, submitted to me in a thesis yesterday; for the pathologist had made the picture and the section with no eye to this point: in it were to be seen dark islands of fibrous tissue, but also many of normal secreting structure; some, indeed, were in the state of hypergranulation which is an early sign of deterioration of the kidney cell, but many more were quite free even from this fault. But in a true granular kidney you will not find these areas in which the secreting tubules and cells are normal. If we turn to an early and acute form of the disease, say a case of acute puerperal nephritis, we shall see acute necrosis in more or less degree almost universal in the renal epithelium. The pathological point is whether the change begins in the cells; the clinical is whether epithelial cells and granular casts appear in the urine. Polyuria as a symptom of chronic nephritis is not characteristic, it is merely the effect of high arterial pressures from any cause, in health or disease. Again, granular kidney generally falls within our first or second period of life; but patients with hyperpiesis without renal disease generally live into the third: their failure is between sixty and seventy, often about sixty-three, whereas most of the kidney patients die between fifty and sixty, or sooner.

To turn to the causes of hyperpiesis; it is sometimes said that I have asserted the high blood-pressure is caused by viscosity of the blood. What I have said is that the cause must be either viscosity of the blood or a narrowing of the calibre of the arteries. These are the only two conditions that can explain it, but which was the agent I never pretended to decide. In the last four or five years the evidence has been against viscosity as the cause. Increase of viscosity seems to depend chiefly upon the number of the red corpuscles. In "congenital cyanosis" the blood-pressure records, though high, are not very high, probably because of compensatory dilation of the vessels. It is becoming more and more probable that the old view is the true one; namely, that high pressure is due to constriction in very large areas, as in the splanchnic or the musculo-cutaneous systems; chiefly in the former. A moderate area of vaso-constriction is easily compensated; but either of these two systems is too vast for such a process, there being no room for the blood to shift elsewhere. Cohnheim made clear this doctrine of pressures in areas. What causes the splanchnic constriction we do not know in the least; some disorder of metabolism is what we say; this, of course, is no explanation at all, but mere verbiage.

I want to lay great stress on this—that the morbid tendency to high pressure is never discovered in its early stages, except by chance. The subjects of it often feel very well, for one thing because there is a greater blood-supply to the brain. Yet the cases are only too common, and in initial stages they are absolutely curable, though they have a tendency to relapse. But if they come under proper control and treatment, they often do not relapse. It takes four or five years, or in moderate cases even ten, to damage the arteries permanently. If the condition is, as usual, undetected, the aorta becomes atheromatous, the heart is overtaxed, and the arteries mechanically spoiled. The patient may not come for advice until he complains that he is short of breath, and in this stage he can no longer be cured: relieved, perhaps; cured, no. Abnormal adaptations have now become established; if by various means you do get his blood-pressure down, you may relieve some of the symptoms arising from cardiac failure; but you may do him harm in other ways, and he knows it, and scouts your assertion that he is "improving." For, as his pressure falls below his abnormal needs, his functions languish, and the circulation is less and less efficient. You must not "cure" him, then, beyond a certain point; and if you try to do it you do your patient harm. But if you can catch him in the early stage, then do all you can by salines, baths, diet alterations, and even cautiously by nitrites if you like, to reduce the blood-pressure to normal.

High blood-pressure has sometimes to be diagnosed from neurasthenia—which we hear spoken of as a symptom of arterio-sclerosis—and from melancholia. But these are totally different diseases, and their occasional association is accidental. I have never happened to see the combination of neurasthenia and hyperpiesis, though I have often seen cases of alleged neurasthenia which proved to be early hyperpiesis. In melancholia, it is true, arterial pressures rule high, but in nature and issue the diseases are different. Moreover, the treatment for these several diseases is totally different, and confusion between them a mischievous mistake.

I may pass quickly by my second class, the toxic cases. Except in lead-poisoning, high pressure is not characteristic of them.

Now I come to our senile class. Over and over again I find excessive pressures spoken of as a senile change, but, as I have told you, the assertion is quite at random. Decrescent atheroma is attended by normal or quasi-normal (elderly) blood-pressures, or even by subnormal—down to 90 mm. Then what is the cause of senile arterio-sclerosis? Certainly I have no explanation. That, although almost universal, it is not essential to old age is now admitted. In a patient of mine who died at the age of eighty-three, the vessels, from head to foot, showed no vestige of atheroma nor of arterio-sclerosis, whether in the large arteries or small. He had had a subphrenic sinus for a long time, due to escape of a stone from the gall-bladder, but no pyrexia, as the capsule of the abscess cavity was dense.

Although in class 1 the arterial damage is a mechanical consequence of hyperpiesis, and of this only, yet, of course, in all forms of the disease, as in health, the arteries are under mechanical strain. But the point is whether the change is due in the first instance to the stress of high blood-pressure alone, or to deterioration of the vessels due to some other cause acting on the nutrition of the vessels themselves, so that they succumb to the normal stresses which good arteries withstand. For seventy or eighty years our vessels are as incessantly beaten by the tides of the pulse as our coasts are pummelled by the waves of the sea; and it is probable that one or many subtle perversions, accumulating slowly over many years, tell heavily at the end of life.

Professor Osler has described the early degeneration of an artery as due to the use of bad material when the tubing was put in: yet I scarcely think all arterio-sclerosis can be explained in this way. It is not fair to accuse the hyperpietic of jerry-built arteries, if, as we have seen, these vessels have been subject for years to pressures equal to those of 180 to 200 mm. of mercury. And is it fair similarly to accuse the arteries of a syphilitic man of being made of poor stuff when they have been constantly bathed in a toxic fluid?

That in some cases there are defects in their original quality may well be; some people, as we see, become senile earlier than others. In two cases lately, at the ages of fifty-one and fifty-five respectively, I have seen all the consequences of utter arterial decay and consequent failure of vital power. It is very likely that such men were born with arteries below standard value; but bad materials are not by any means always at the bottom of these diseases. Even senile atheroma may be due to disorders of metabolism of an obscure kind which have been insensibly corroding the vessels for many a year.

The gentleman whose arteries were in such perfect condition at eighty-three had had the sinus from his abscess for twenty-two years. There is some evidence—not much, but enough to make us think—that an issue may postpone or prevent some toxic corrosions of this kind.

And this leads me to a last word on venesection. It is always difficult to prescribe a far-reaching and somewhat ascetic regimen for these patients, and if venesection is to be added one needs the co-operation of a practitioner whose influence in the family carries weight. In cases of relatively young men with very high and obstinate blood-pressures I think that venesection has an indubitably good effect. Thus we may guess why our forefathers were bled in spring and autumn. Physicians in the seventeenth and early eighteenth centuries did not bleed promiscuously; they knew which patients to bleed, and how it did them good. Sangrado-ism set in somewhat later under the Brown and Broussais influences. I have had the most grateful letters from hyperpietic patients telling me that for some years they had not felt so well as for some months after each time they had been bled.

Finally, it is no rare coincidence for hyperpiesis to arise in persons already the subjects of senile atheroma; and, if not detected and neutralised, to cut through the senile process, and so greatly to accelerate the fatal issue. Happily, in these cases the hyperpiesis is usually incidental and so manageable, and by its reduction the patient is brought back to the state of simple decrescent atheroma.

